# Effects of genetic polymorphisms on methotrexate levels and toxicity in Chinese patients with acute lymphoblastic leukemia

**DOI:** 10.1097/BS9.0000000000000142

**Published:** 2022-11-11

**Authors:** Qishan Hao, Yang Song, Qiuyun Fang, Yani Lin, Long Chen, Xiaodan Wang, Ping Zhang, Zhe Wang, Xiaoyuan Gong, Kaiqi Liu, Qinghua Li, Zheng Tian, Min Wang, Jianxiang Wang, Yingchang Mi

**Affiliations:** a State Key Laboratory of Experimental Hematology, National Clinical Research Center for Blood Diseases, Haihe Laboratory of Cell Ecosystem, Institute of Hematology & Blood Diseases Hospital, Chinese Academy of Medical Sciences & Peking Union Medical College, Tianjin, China; b SINO-US Diagnostics Lab, Tianjin, China.

**Keywords:** Acute lymphoblastic leukemia, Concentration, Methotrexate, Polymorphism, Toxicity

## Abstract

Methotrexate (MTX) has an antitumor effect when used for the treatment of acute lymphoblastic leukemia (ALL). This study aims at evaluating the associations between 14 polymorphisms of six genes involved in MTX metabolism with serum MTX concentration and toxicity accompanying high-dose MTX. Polymorphisms in 183 Chinese patients with ALL were analyzed using TaqMan single nucleotide polymorphism genotyping assay. The serum MTX concentration was determined using homogeneous enzyme immunoassay. MTX-related toxicities were also evaluated. Renal toxicity was significantly associated with higher serum MTX concentrations at 24, 48, and 72 hours, and MTX elimination delay (*P* = 0.001, *P* < 0.001, *P* < 0.001, and *P* < 0.001, respectively), whereas *SLCO1B1* rs4149056 was associated with serum MTX concentrations at 48 and 72 hours, and MTX elimination delay in candidate polymorphisms (*P* = 0.014, *P* = 0.019, and *P* = 0.007, respectively). *SLC19A1* rs2838958 and rs3788200 were associated with serum MTX concentrations at 24 hours (*P* = 0.016, *P* = 0.043, respectively). *MTRR* rs1801394 was associated with serum MTX concentrations at 72 hours (*P* = 0.045). Neutropenia was related to *SLC19A1* rs4149056 (odds ratio [OR]: 3.172, 95% confidence interval [CI]: 1.310–7.681, *P* = 0.011). Hepatotoxicity was associated with *ABCC2* rs2273697 (OR: 3.494, 95% CI: 1.236–9.873, *P* = 0.018) and *MTRR* rs1801394 (OR: 0.231, 95% CI: 0.084–0.632, *P* = 0.004). Polymorphisms of *SLCO1B1, SLC19A1, ABCC2*, and *MTRR* genes help predict higher risk of increased MTX levels or MTX-related toxicities in adult ALL patients.

## 1. INTRODUCTION

Acute lymphoblastic leukemia (ALL) is a hematological malignancy characterized by blast lymphocyte proliferation in the bone marrow, peripheral blood, and extramedullary sites.^1^ Compared with the high-cure rate of childhood ALL, the outcome of adult ALL is poor.

**Figure 1.**
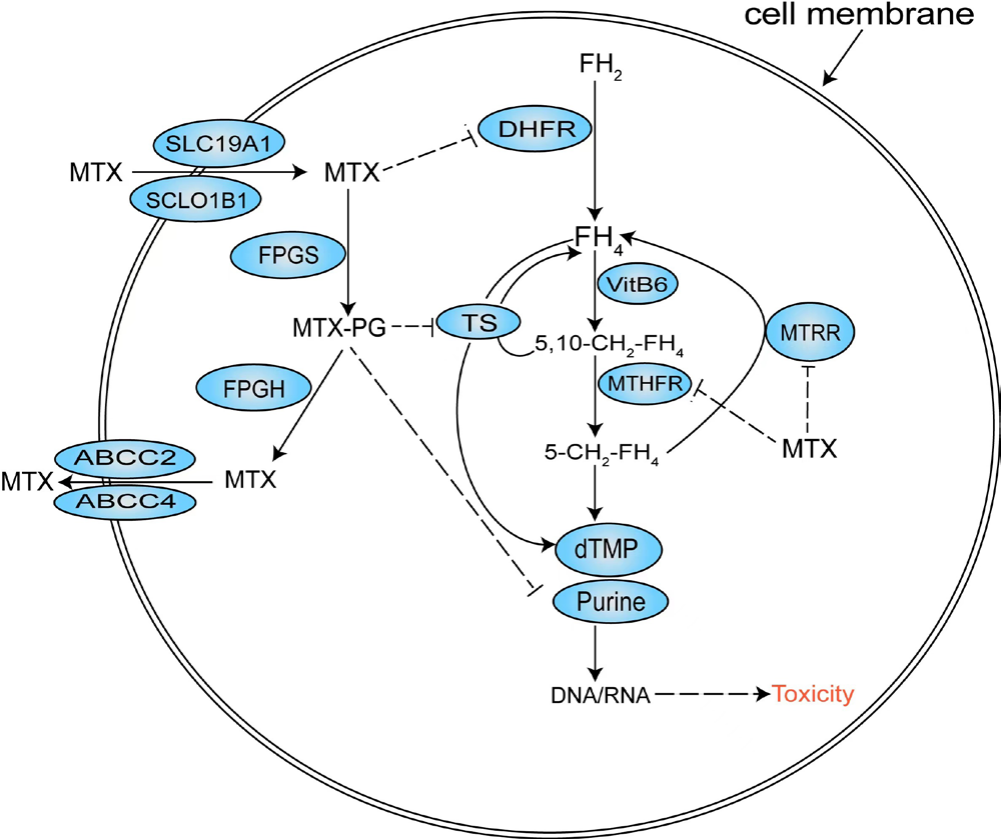
Metabolic pathway of folate and MTX. 5,10-CH2-FH4, 5,10-methylenetetrahydrofolate; 5-CH2-FH4, 5-methylenetetrahydrofolate; 6MP, 6-mercaptopurine; ABCC, ATP-binding cassette, sub-family C; DHFR, dihydrofolate reductase; dTMP, deoxythymidylate; FH_2_, dihydrofolate; FH_4_, tetrahydrofolate; FPGH, folylpolyglutamate hydrolase; FPGS, folylpolyglutamate synthetase; MTHFR, methylenetetrahydrofolate reductase; MTRR, methionine synthase reductase; MTX, methotrexate; MTX-PG, methotrexate-polyglutamate; NUDT15, nucleoside diphosphate-linked moiety X motif 15; SLC19A1, solute carrier 19A1; SLCO1B1, solute carrier organic anion transporter 1B1; TPMT, thiopurine S-methyltransferase; TS, thymidylate synthase; VitB6, vitamin B6.

Methotrexate (MTX) is an essential antitumor drug for ALL and a folate antagonist that can competitively inhibit dihydrofolate reductase, which converts dihydrofolate into tetrahydrofolate. The lack of tetrahydrofolate impairs purine and thymidine synthesis, which in turn impairs DNA replication and eventually results in cell death.^2^ Additionally, MTX inhibits other enzymes involved in folate metabolism, including methylenetetrahydrofolate reductase (MTHFR) and methionine synthase reductase (MTRR). MTX enters the cells by binding to solute carrier organic anion transporter family member 1B1 (SLCO1B1) or solute carrier 19A1 (SLC19A1), while being exported from the cells through various ATP-binding cassette (ABC) transporters (ABCC1-5 and ABCG1-2) (Fig. 1).^3–5^ The MTX distribution, bioavailability, and clearance in the body show marked differences, independent of disease-type, and administration routes.^6^ However, an interpatient variability in response to MTX is associated with variations in genes encoding enzymes and membrane transporter proteins involved in the MTX metabolism. Patients with ALL receiving high-dose MTX (HD-MTX) frequently suffer from mucositis, gastrointestinal toxicity, hematologic toxicity, hepatoxicity, and nephrotoxicity.^7,8^ MTX-related toxicities limit MTX dosage and result in treatment delay. Thus, the potential tolerance of patients with ALL to MTX should be determined at the pretreatment stage.

Previous studies have investigated the association between single nucleotide polymorphisms (SNPs) of genes in the MTX/folate metabolic pathway and MTX levels and MTX-related toxicity; however, the results are contradictory (see Supplemental Table 1, http://links.lww.com/BS/A51). The conflicting outcomes may be caused by small or varying populations, different treatment regimens and nonuniform toxicity standards. Moreover, most previous studies were conducted in children with ALL. Therefore, the current study aimed at exploring the association between 14 SNPs of six genes involved in MTX metabolism with serum MTX concentrations and HD-MTX-related toxicities in adolescent and adult Chinese patients with ALL.

## 2. MATERIALS AND METHODS

### 2.1. Patient and clinical data

A total of 183 patients with ALL who received HD-MTX treatment and were ≥14 years old were included in this retrospective study. All patients were newly diagnosed and treated in the Leukemia Department of the Institute of Hematology & Blood Diseases Hospital, Chinese Academy of Medical Sciences & Peking Union Medical College (Tianjin, China) from September 2015 to December 2019. The study was approved by the Ethics Committee of the hospital (No. HG2021038-EC-1). Written informed consent was obtained from all patients or their guardians.

According to the ChiCTR-TNC-09000397 protocol, the MTX dose was 2 g/m^2^ in patients with Philadelphia chromosome-positive ALL (Ph^+^ ALL) and 3 g/m^2^ in those with Philadelphia chromosome-negative ALL (Ph^−^ ALL). About one-third of the total MTX dose was infused within 1 hour, the remainder administered as a 23-hour continuous intravenous infusion. Leucovorin rescue therapy was started at a dose of 7.5% of the MTX dose and 36 hours after the start of MTX administration. The total leucovorin delivered was divided into 6 to 8 doses at 6-hour intervals (the first dose was 50 mg) until the serum MTX concentration was <0.3 μmol/L. The serum MTX concentration was measured daily at 24, 48, and 72 hours after the start of MTX administration and was determined by homogeneous enzyme immunoassay (Siemens Viva-E Drug Test System, Erlangen, Bavaria, Germany) using the MTX detection kit (Siemens, Newark, DE, USA) in the hospital’s Clinical Pharmacy Laboratory, with a safe value of 0.3 μmol/L. MTX elimination delay was defined as MTX concentration of >1.0 μmol/L at 48 hours and >0.3 μmol/L at 72 hours.

MTX-related toxicities, including hematologic, hepatic and renal, were evaluated according to the Common Terminology Criteria for adverse events version 5.0 (CTCAE v.5.0).^9^ The highest toxicity grade was observed and registered in each patient within 2 weeks of starting the MTX medication. Toxicities were assessed based on clinical parameters, such as white blood cell, absolute neutrophil and platelet counts, and hemoglobin, alanine aminotransferase (ALT), aspartate aminotransferase (AST), bilirubin, and creatinine levels. An increase or decrease in one or more grades of these items from their initial values was considered as toxicity.

### 2.2. DNA extraction and genotyping assay

Mononuclear cells were isolated from the bone marrow of newly diagnosed patients on a Ficoll density gradient (QuatoBio, Beijing, China). Genomic DNA was extracted using the QIAamp DNA Mini Kit (Qiagen, Hilden, NRW, Germany), according to the manufacturer’s protocol. Nanodrop 2000 (Thermo Fisher Scientific, Waltham, MA, USA) was used to measure DNA concentration and purity. DNA samples were stored at −20°C. A total of 14 SNPs of six genes involved in MTX/folate metabolism (*SLCO1B1, SLC19A1, ABCC2, ABCC4, MTRR, MTHFR*) were selected. Alleles were determined using TaqMan SNP Genotyping Assay on a QuantStudio 5 real-time polymerase chain reaction system (Applied Biosystems, Foster City, CA, USA) following the user’s manual. The total volume per well was 10 μL, containing 5 μL TaqPath ProAmp Master Mix (1×), 0.5 μL TaqMan probe (20×), 2 μL DNA (10–20 ng/μL), and 2.5 μL double-distilled water. Both TaqMan probe and TaqPath ProAmp Master Mix were bought from Thermo Fisher Scientific (see Supplemental Table 2, http://links.lww.com/BS/A52). The polymerase chain reaction conditions were as follows: 60°C for 30 seconds, 95°C for 5 minutes (95°C for 15 seconds, 60°C for 60 or 90 seconds) for 45 cycles, and 60°C for 30 seconds. An automatic allele calling was carried out using the QuantStudio Design and Analysis Software v1.4.1 (Thermo Fisher Scientific, Waltham, MA, USA).

### 2.3. Statistical analysis

The SPSS statistical software version 25.0 (IBM, Armonk, NY, USA) was employed for statistical analysis. Hardy-Weinberg equilibrium (HWE) of genotype frequencies was assessed in 183 patients using the chi-squared test. Categorical variables (such as MTX elimination delay) were assessed by chi-squared or Fisher’s exact test. Continuous variables (such as the serum MTX concentration) were expressed as median and interquartile range (IQR). The Mann-Whitney *U* test was carried out to analyze the associations between serum MTX concentration and SNPs and MTX-related toxicity. The association between MTX-related toxicity and elimination delay was evaluated using Fisher’s exact test. The association between SNPs and MTX elimination delay, as well as MTX-related toxicity, was assessed using logistic regression analysis and presented as odds ratio (ORs) and 95% confidence interval (CI). In logistic regression analysis, gender, age, ethnicity, immunophenotype, MTX dose, body surface area, and tyrosine kinase inhibitors (TKI) were included as covariates. A *P* value of <0.05 was considered statistically significant.

## 3. RESULTS

### 3.1. Patient characteristics and genotyping

The characteristics of the 183 patients with ALL are summarized in Table 1. Briefly, this study included 102 male (55.74%) and 81 female (44.26%) patients. A total of 57 patients (31.15%) were Ph^+^ ALL, 126 (68.85%) were Ph^−^ ALL, 158 (86.34%) were B-lineage ALL (B-ALL) and 25 (13.66%) were T-lineage ALL (T-ALL). The median age of the cohort was 32 (range: 14–66) years. Among the patients with Ph^+^ ALL, 32 (56.14%) received imatinib treatment, while the other 25 (43.86%) received dasatinib. The median MTX concentrations at 24, 48, and 72 hours were 52.92 (IQR: 31.32–75.60), 0.62 (IQR: 0.28–1.95), and 0.15 (IQR: 0.07–0.62) μmol/L, respectively. The frequency of MTX elimination delay was 43.72%. The results of genotypes in all patients are shown in Table 2. Genotype frequencies of 12 SNPs were in the HWE, except for *ABCC2* rs2273697 and *MTHFR* rs1801133. Table 3 summarizes MTX-related toxicities. Leukopenia was the most common with a frequency of 78.14%, followed by neutropenia (67.76%) and thrombocytopenia (57.92%). The frequency of anemia and increased creatinine level was 56.28% and 25.68%, respectively.

**Table 1:** Characteristics of 183 study subjects

Characteristics	
Sex, n (%)	
Male	102 (55.74)
Female	81 (44.26)
Age at diagnosis [Median (range)], years	32 (14–66)
Ethnicity, n (%)	
Han	177 (96.72)
Hui	2 (1.09)
Manchu	2 (1.09)
Mongol	1 (0.55)
Daur	1 (0.55)
Immunologic subtype, n (%)	
B-ALL	158 (86.34)
T-ALL	25 (13.66)
Philadelphia chromosome, n (%)	
Negative	126 (68.85)
Positive	57 (31.15)
Tyrosine kinase inhibitor, n (%)	
Imatinib	32 (56.14)
Dasatinib	25 (43.86)
Body surface area (range), m^2^	1.70 (1.29–2.35)
Median MTX concentration at 24 h (IQR), μmol/L	52.92 (31.32–75.60)
Median MTX concentration at 48 h (IQR), μmol/L	0.62 (0.28–1.95)
Median MTX concentration at 72 h (IQR), μmol/L	0.15 (0.07–0.62)
MTX concentration >1 μmol/L at 48 h, n (%)	67 (36.61)
MTX concentration >0.3 μmol/L at 72 h, n (%)	73 (39.89)
MTX elimination delay, n (%)	80 (43.72)

B-ALL, B-lineage acute lymphoblastic leukemia; IQR, interquartile range; MTX, methotrexate; T-ALL, T-lineage acute lymphoblastic leukemia.

**Table 2:** Genotype frequency of SNPs

SNPs	n (%)	*P* *	SNPs	n (%)	*P*
*SLCO1B1* rs4149056 T>C		0.549	*ABCC2* rs717620 C>T		0.426
TT	139 (75.96)		CC	119 (65.03)	
TC	42 (22.95)		CT	55 (30.05)	
CC	2 (1.09)		TT	9 (4.92)	
*SLCO1B1* rs2306283 A>G		0.424	*ABCC2* rs2273697 G>A		0.034
AA	11 (6.01)		GG	153 (83.61)	
AG	76 (41.53)		GA	26 (14.21)	
GG	96 (52.46)		AA	4 (2.19)	
*SLCO1B1* rs4149081 G>A		0.721	*ABCC4* rs9302061 C>T		0.258
GG	62 (33.88)		CC	74 (40.44)	
GA	87 (47.54)		CT	79 (43.17)	
AA	34 (18.58)		TT	30 (16.39)	
*SLCO1B1* rs11045879 T>C		0.739	*ABCC4* rs7317112 A>G		0.056
TT	58 (31.69)		AA	106 (57.92)	
TC	92 (50.27)		AG	60 (32.79)	
CC	33 (18.03)		GG	17 (9.29)	
*SLC19A1* rs2838958 G>A		0.743	*MTRR* rs1801394 A>G		0.911
GG	37 (20.22)		AA	115 (62.84)	
GA	93 (50.82)		AG	59 (32.24)	
AA	53 (28.96)		GG	9 (4.92)	
*SLC19A1* rs3788200 A>G		0.412	*MTHFR* rs1801131 A>C		0.550
AA	34 (18.58)		AA	134 (73.22)	
AG	96 (52.46)		AC	44 (24.04)	
GG	53 (28.96)		CC	5 (2.73)	
*ABCC2* rs3740065 A>G		0.324	*MTHFR* rs1801133 C>T		0.026
AA	66 (36.07)		CC	38 (20.77)	
AG	93 (50.82)		CT	74 (40.44)	
GG	24 (13.11)		TT	71 (38.80)	

* Hardy-Weinberg equilibrium (HWE) of genotype frequencies was assessed using Chi-square test.

SNPs, single nucleotide polymorphisms.

**Table 3:** MTX-related toxicity and MTX serum levels

		24 h (μmol/L)	*P* *	48 h (μmol/L)	*P* *	72 h (μmol/L)	*P* *	MTX elimination delay		*P* †
Adverse events	n (%)	Median (IQR)		Median (IQR)		Median (IQR)		Yes	No	
Leukopenia										
Yes	143 (78.14)	50.76 (30.60–72.00)		0.64 (0.28–2.70)		0.17 (0.06–0.71)		62 (75.0)	81 (78.64)	
No	40 (21.86)	56.52 (32.31–94.50)	0.423	0.53 (0.28–1.41)	0.857	0.14 (0.08–0.43)	0.956	18 (25.0)	22 (21.36)	0.859
Neutropenia										
Yes	124 (67.76)	50.76 (30.87–71.28)		0.58 (0.27–2.63)		0.14 (0.06–0.71)		52 (65.0)	72 (69.90)	
No	59 (32.24)	54.72 (31.32–92.88)	0.148	0.73 (0.33–1.49)	0.370	0.19 (0.08–0.51)	0.309	28 (35.0)	31 (30.10)	0.525
Anemia										
Yes	103 (56.28)	55.44 (33.84–88.56)		0.77 (0.28–3.30)		0.23 (0.07–1.03)		50 (62.5)	53 (51.46)	
No	80 (43.72)	45.72 (28.89–71.46)	0.212	0.53 (0.29–1.21)	0.235	0.12 (0.06–0.44)	0.119	30 (37.5)	50 (48.54)	0.176
Thrombocytopenia										
Yes	106 (57.92)	54.90 (30.60–76.14)		0.66 (0.28–3.30)		0.17 (0.06–0.80)		47 (58.75)	59 (57.28)	
No	77 (42.08)	46.08 (33.66–84.24)	0.834	0.56 (0.31–1.39)	0.595	0.15 (0.08–0.42)	0.825	33 (41.25)	44 (42.71)	0.881
Increased ALT										
Yes	36 (19.67)	46.44 (30.24–68.13)		0.80 (0.29–2.63)		0.19 (0.04–0.77)		17 (21.25)	19 (18.45)	
No	143 (80.33)	54.72 (32.04–92.88)	0.522	0.58 (0.28–1.88)	0.644	0.15 (0.08–0.61)	0.642	63 (78.75)	84 (81.55)	0.709
Increased AST										
Yes	26 (14.21)	45.72 (30.06–84.78)		0.89 (0.27–2.79)		0.28 (0.05–0.83)		13 (16.25)	13 (12.62)	
No	157 (85.79)	54.00 (31.86–80.28)	0.842	0.62 (0.29–1.92)	0.778	0.15 (0.07–0.62)	0.845	67 (83.75)	90 (87.38)	0.527
Increased bilirubin										
Yes	35 (19.13)	60.48 (32.40–99.36)		0.86 (0.28–4.26)		0.22 (0.06–0.67)		19 (23.75)	16 (15.53)	
No	148 (80.87)	49.68 (30.78–71.91)	0.323	0.57 (0.28–1.80)	0.435	0.15 (0.07–0.62)	0.946	61 (76.25)	87 (84.47)	0.187
Increased creatinine										
Yes	47 (25.68)	66.24 (44.28–105.84)		4.44 (2.88–6.96)		1.55 (0.79–3.00)		45 (56.25)	2 (1.94)	
No	136 (74.32)	45.00 (27.72–66.51)	0.001	0.40 (0.27–6.96)	<0.001	0.10 (0.05–0.24)	<0.001	35 (43.75)	101 (98.06)	<0.001

* *P* value was estimated by Mann-Whitney *U* test.

† *P* value was estimated by Fisher’s exact test.

ALT, alanine aminotransferase; AST, aspartate aminotransferase; CI, confidence interva; IQR, interquartile range; MTX, methotrexate; OR, odds ratio.

### 3.2. MTX concentration, MTX elimination delay, and MTX-related toxicity

The associations among MTX-related toxicity, serum MTX concentrations, and MTX elimination delay were evaluated using the Mann-Whitney *U* test or Fisher’s exact test. The results are shown in Table 3. Patients with increased creatinine levels had higher serum MTX concentrations at 24, 48, and 72 hours compared with those without increased levels (*P* = 0.001, *P* < 0.001, and *P* < 0.001, respectively). Additionally, the frequency of renal toxicity was significantly higher in patients with than those without MTX elimination delay (*P* < 0.001).

### 3.3. MTX concentration, MTX elimination delay, and SNPs

**Figure 2.**
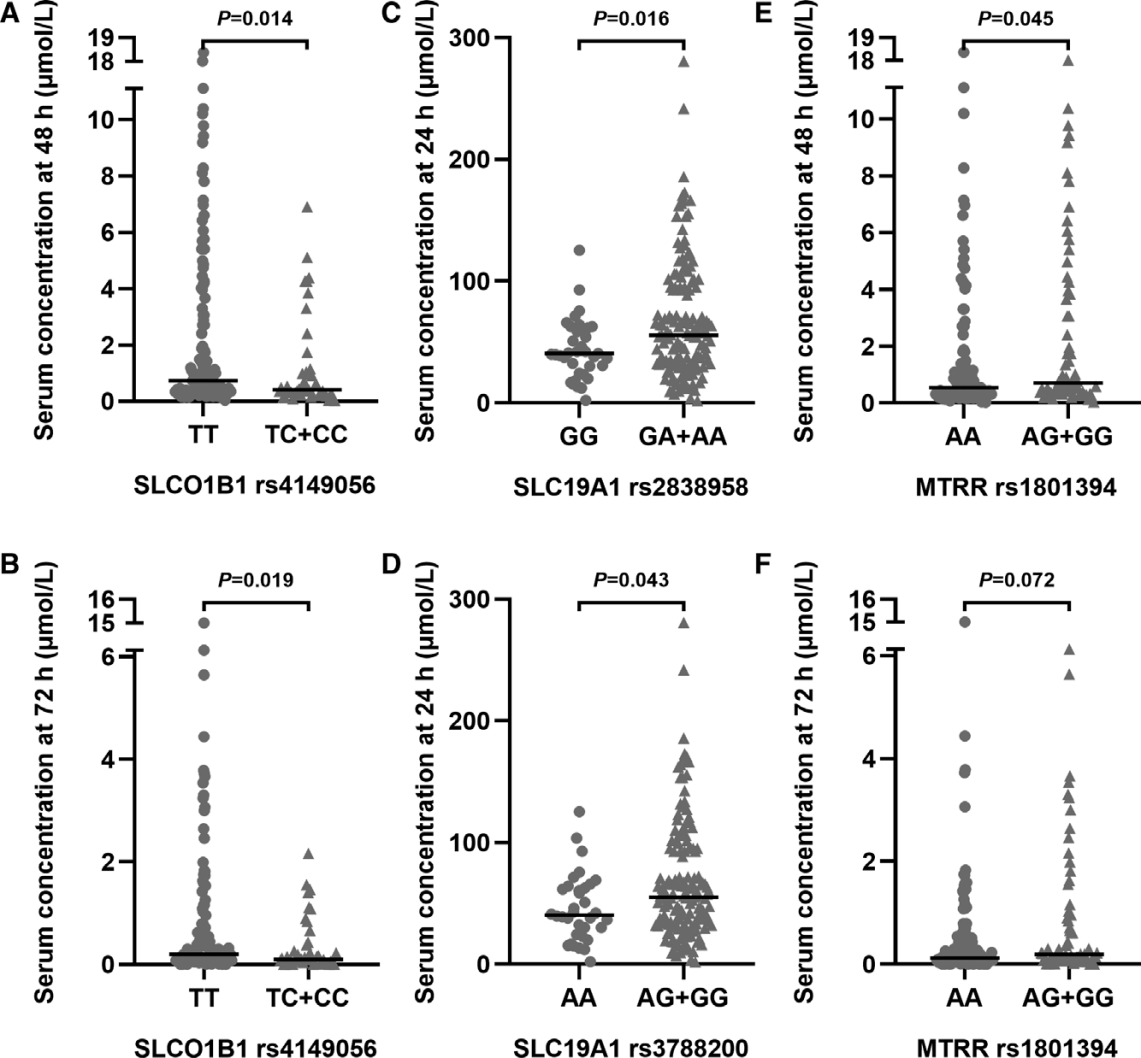
Genotype and serum MTX levels. (A, B) Serum MTX concentrations of patients with *SLCO1B1* rs4149056 wild-type (TT) and variant (TC+CC) at 48 and 72 hours, respectively; (C) Serum MTX concentrations of patients with *SLC19A1 rs2838958* wild-type (GG) and variant (GA+AA) at 24 h; (D) Serum MTX concentrations of patients with *SLC19A1* rs3788200 wild-type (AA) and variant (AG+GG) at 24 h; (E, F) Serum MTX concentrations of patients with *MTRR* rs1801394 wild-type (AA) and variant (AG+GG) at 48 and 72 hours, respectively. Each horizontal line in the figure represents the median serum MTX concentration. *P* value was estimated by Mann-Whitney *U* test. MTX, methotrexate.

Next, we explored the associations of SNPs with serum MTX concentration and MTX elimination delay using the Mann-Whitney *U* test and logistic regression analysis (see Supplemental Table 3, http://links.lww.com/BS/A52). Since dasatinib may affect MTX clearance,^10^ TKI was included as a covariate in the logistic regression analysis. The serum MTX concentrations of patients with *SLCO1B1* rs4149056 TT genotype at 48 and 72 hours were significantly higher than those with the TC or CC genotype (*P* = 0.014 and *P* = 0.019, respectively; Fig. 2A and 2B). Patients with *SLC19A1* rs2838958 GA or AA genotype had higher serum MTX concentrations at 24 hours than those with GG genotype (*P* = 0.016; Fig. 2C). Additionally, patients with *SLC19A1* rs3788200 AG or GG genotype had higher serum MTX concentrations at 24 hours than those with AA genotype (*P* = 0.043; Fig. 2D). Serum MTX levels at 48 hours were higher in patients with *MTRR* rs1801394 AG or GG genotype (*P* = 0.045; Fig. 2E). However, no significantly statistical difference was detected in the MTX serum concentrations at 72 hours between patients with *MTRR* rs1801394 G allele (AG or GG genotype) and AA genotype (*P* = 0.072; Fig. 2F). Moreover, MTX elimination delay was less in patients with *SLC19A1* rs4149056 TC or CC genotype (OR 0.319, 95% CI: 0.138–0.736, *P* = 0.007).

### 3.4. MTX-related toxicities and SNPs

We analyzed the effects of SNPs on MTX-related toxicity using logistic regression analysis (see Supplemental Table 4, http://links.lww.com/BS/A52). Patients with *SLC19A1* rs4149056 TC or CC genotype had a higher risk for neutropenia (OR: 3.172, 95% CI: 1.310–7.681, *P* = 0.011). Hepatotoxicity (increased AST) was associated with *ABCC2* rs2273697 A allele (GA or AA genotype) (OR: 3.494, 95% CI: 1.236–9.873, *P* = 0.018). Furthermore, patients with *MTRR* rs1801394 AG or GG genotype experienced less hepatotoxicity (increased bilirubin) (OR: 0.231, 95% CI: 0.084–0.632, *P* = 0.004).

## 4. DISCUSSION

MTX is a key antitumor drug widely used for the treatment of ALL. The response to MTX greatly varies among patients receiving HD-MTX treatment. In the current retrospective study, we analyzed the association of candidate SNPs with pharmacokinetics and toxicities of HD-MTX in 183 Chinese patients with ALL.

First, we analyzed the associations between serum MTX concentrations, MTX elimination delay, and MTX-related toxicities. As in previous reports,^11,12^ the present study showed that renal toxicity was significantly associated with high MTX concentrations and high incidence of MTX elimination delay. Earlier research has demonstrated that hyperbilirubinemia was associated with high MTX concentrations.^12^ However, effects of MTX delayed elimination and high MTX concentrations on hematological profiles and liver function were not found in our study. In general, we support the use of the MTX concentration as an objective and efficacious marker of MTX-related toxicities.

SLCO1B1 transporter is encoded by the *SLCO1B1* gene, which transports MTX into the cells.^4^ Genetic variants of *SLCO1B1* have been reported to affect MTX pharmacokinetics. Two variants in *SLCO1B1*, rs4149081 and rs11045879, have been identified as being associated with MTX-related toxicity and MTX clearance in a genome-wide analysis study.^13^ Ramsey et al. confirmed that the *SLCO1B1* variants, rs4149056 and rs2306283, were predictors of MTX clearance in patients with ALL.^14,15^ In the present study, associations between *SLCO1B1* rs4149056 and MTX serum level and elimination delay were established, a result similar to that of den Hoed et al.^16^ Additionally, an increased risk of neutropenia was associated with rs4149056 variant (TC or CC genotype). An earlier study in children with juvenile idiopathic arthritis suggested that rs4149056 variant (TC or CC genotype) was significantly associated with MTX gastrointestinal toxicity and TT genotype was a risk factor for developing hepatotoxicity.^17^ However, associations of rs2306283, rs4149081, and rs11045879 with MTX metabolism were not established. Differences in these findings indicate that genetic polymorphisms may affect *SLCO1B1*’s function through different SNPs simultaneously. Moreover, Ramsey et al. reported that dasatinib delayed MTX clearance through the mediation of transporter SLCO1B1.^10^ There have also been recent case reports of delayed MTX clearance caused by concomitant use of imatinib.^18,19^ The TKI mechanisms affecting MTX metabolism need to be studied further.

MTX can also be transported into cells through the cell membrane by binding to the membrane protein encoded by *SLC19A1* (also known as *RFC1*).^3^ Therefore, the influence of *SLC19A1* polymorphisms on MTX metabolism remains controversial. In the current study, both *SLC19A1* rs2838958 and *SLC19A1* rs3788200 mutant alleles were significantly associated with higher serum MTX levels. These findings were not supported by previous studies.^20,21^ A study conducted in Slovenia suggested that *SLC19A1* rs2838958 AA genotype results in a significant level of mucositis; however, no association with leukopenia was reported.^22^ Furthermore, our results indicated that SNPs of *SLC19A1* have no effect on MTX toxicity. These inconsistent results may be explained by the differences in study populations and treatment regimens.

The ABC transporter family is one of the largest membrane transporters mediating the export of MTX from cells.^5^ *ABCC2* polymorphisms, especially rs717620, have been confirmed as contributing to the variability of MTX kinetics.^23^ However, the influence of *ABCC2* rs717620 on MTX levels, MTX clearance, and hematological toxicity was at variance with the results of several clinical studies.^24–27^ The effects of *ABCC2* rs717620 on MTX metabolism were not observed in our study. Neither MTX serum level nor MTX elimination delay was associated with *ABCC2* rs3740065, as reported by Liu et al.^26^ Furthermore, we did not find any association between *ABCC2* rs3740065 and MTX toxicity. Conversely, Lopez-Lopez et al, in a cohort of 151 children with ALL, suggested that the *ABCC2* rs3740065 G allele was significantly associated with high MTX concentration at 72 hours and an elevated risk of MTX-related toxicity.^20^ Although no genetic variant of *ABCC2* rs2273697 was found to have an impact on serum MTX concentration and MTX elimination delay as previously reported,^23,28^ patients with the *ABCC2* rs2273697 A allele had a higher risk of developing hepatoxicity (increased AST). Genetic variability in *ABCC4* rs9302061 and rs7317112 did not influence MTX metabolism, a result consistent with that of a previous study.^20^ However, den Hoed *et al*.^16^ carried out a study on *ABCC4* rs7317112 in Dutch children with ALL and indicated that the AA genotype was related to mucositis and high MTX plasma levels at 48 h.

Furthermore, we demonstrated that *MTRR* rs1801394 G allele (AG or GG genotype) was associated with high serum MTX concentrations. Nonetheless, a study from the Netherlands found that *MTRR* rs1801394 AA genotype was associated with higher MTX levels.^16^ *MTRR* rs1801394 polymorphism had no impact on MTX elimination delay and MTX toxicity in the current study. Associations of *MTRR* rs1801394 with plasma MTX level and MTX-induced toxicity were not observed in Turkish children with ALL.^29^ Furthermore, the association of this polymorphism with MTX-related toxicity was not detected in a study of 162 Chinese patients with rheumatoid arthritis treated with MTX.^30^ Further studies are warranted to determine the effects of polymorphism on MTX metabolism.

The decreased activity of MTHFR enzyme may affect MTX efficacy and toxicity. Two variants, rs1801131 and rs1801133, in *MTHFR* are the most extensively investigated polymorphisms in genetic variation studies of patients with ALL.^4^ Both *MTHFR* rs1801131 AC or CC genotype and AA genotype were identified as risk factors for MTX-related toxicities in several studies.^7,8^ The majority of studies have revealed that *MTHFR* rs1801133 CT or TT genotype had a risk of high MTX plasma concentration, MTX elimination delay and MTX-related toxicity.^8,31^ Several other studies have suggested that the *MTHFR* rs1801133 CC genotype had a high risk of MTX-related toxicity.^7,32,33^ Han et al claimed that *MTHFR* rs1801133 T allele and rs1801131 AA genotype were risk factors for hematopoietic toxicity in a study of 157 adult Chinese patients with hematological malignancies.^34^ Several previous studies had shown neither rs1801131 nor rs1801133 polymorphisms in *MTHFR* were significantly associated with MTX-related toxicity and delayed MTX clearance in pediatric patients with ALL.^29,35,36^ Unfortunately, associations of *MTHFR* rs1801131 and rs1801133 polymorphisms with MTX metabolism are not yet established. Given the cumulative evidence, whether SNPs of *MTHFR* can be used as predictors of MTX concentration and MTX-related toxicity remains controversial.

The results of studies on the association between genetic polymorphisms and MTX metabolism are inconsistent, which could be attributed to small sample sizes, different toxicity criteria, varied treatment protocols, and racial differences. The advantages of the current study include unified diagnostic criteria, treatment regimens, and quantifiable and objective toxicity data from a single center. Nonetheless, the retrospective study was limited to MTX-related toxicities, which included hematologic toxicity, hepatotoxicity, and renal dysfunction but did not analyze other common drug side effects, such as oral mucositis and vomiting or diarrhea. The current findings require further substantiation.

## 5. CONCLUSION

The current results indicated that MTX serum levels forecast MTX-related toxicity. Genetic polymorphisms involved in the MTX metabolism pathways, such as *SLCO1B1* rs4149056, *SLC19A1* rs2838958, *SLC19A1* rs3788200, and *MTRR* rs1801394, were associated with MTX serum levels in adult ALL. Moreover, genetic polymorphisms, such as *SLC19A1* rs4149056, *ABCC2* rs2273697, and *MTRR* rs1801394, were related to MTX-related toxicities.

## ACKNOWLEDGMENTS

We thank the patients for their participation and colleagues in the clinical pharmacy laboratory for their contribution to this work.
